# Study on the Adaptability of FBG Sensors Encapsulated in CNT-Modified Gel Material for Asphalt Pavement

**DOI:** 10.3390/gels11080590

**Published:** 2025-07-31

**Authors:** Tengteng Guo, Xu Guo, Yuanzhao Chen, Chenze Fang, Jingyu Yang, Zhenxia Li, Jiajie Feng, Jiahua Kong, Haijun Chen, Chaohui Wang, Qian Chen, Jiachen Wang

**Affiliations:** 1 School of Civil Engineering and Transportation, North China University of Water Resources and Electric Power, Zhengzhou 450045, China; guotth@ncwu.edu.cn (T.G.); 17538255803@163.com (X.G.); fangchenze@ncwu.edu.cn (C.F.); yangjingyu@ncwu.edu.cn (J.Y.); zhenxiali2009@ncwu.edu.cn (Z.L.); 18027459877@163.com (J.F.); 18539291281@163.com (J.K.); chenhaijun@ncwu.edu.cn (H.C.); 2 Henan Province Engineering Technology Research Center of Environment Friendly and High-Performance Pavement Materials, Zhengzhou 450045, China; 3 Technology Innovation Center of Henan Transport Industry of Utilization of Solid Waste Resources in Traffic Engineering, North China University of Water Resources and Electric Power, Zhengzhou 450045, China; 4 Henan Province University-Enterprise Research and Development Center for Green, Low-Carbon and High-Performance Road Materials, Zhengzhou 450045, China; 5 School of Highway, Chang’an University, Xi’an 710064, China; wchh0205@chd.edu.cn (C.W.); chenqian@chd.edu.cn (Q.C.); 6 Department of Electrical Engineering, College of Science and Technology, University of Nottingham Ningbo China, Ningbo 315100, China; ssyjw19@nottingham.edu.cn

**Keywords:** asphalt pavement, CNT-modified gel material, fiber grating sensor, calibration test, performance study

## Abstract

To prolong the service life of asphalt pavement and reduce its maintenance cost, a fiber Bragg grating (FBG) sensor encapsulated in carboxylated carbon nanotube (CNT-COOH)-modified gel material suitable for strain monitoring of asphalt pavement was developed. Through tensile and bending tests, the effects of carboxylated carbon nanotubes on the mechanical properties of gel materials under different dosages were evaluated and the optimal dosage of carbon nanotubes was determined. Infrared spectrometer and scanning electron microscopy were used to compare and analyze the infrared spectra and microstructure of carbon nanotubes before and after carboxyl functionalization and modified gel materials. The results show that the incorporation of CNTs-COOH increased the tensile strength, elongation at break, and tensile modulus of the gel material by 36.2%, 47%, and 17.2%, respectively, and increased the flexural strength, flexural modulus, and flexural strain by 89.7%, 7.5%, and 63.8%, respectively. Through infrared spectrum analysis, it was determined that carboxyl (COOH) and hydroxyl (OH) were successfully introduced on the surface of carbon nanotubes. By analyzing the microstructure, it can be seen that the carboxyl functionalization of CNTs improved the agglomeration of carbon nanotubes. The tensile section of the modified gel material is rougher than that of the pure epoxy resin, showing obvious plastic deformation, and the toughness is improved. According to the data from the calibration experiment, the strain and temperature sensitivity coefficients of the packaged sensor are 1.9864 pm/μm and 0.0383 nm/°C, respectively, which are 1.63 times and 3.61 times higher than those of the bare fiber grating. The results of an applicability study show that the internal structure strain of asphalt rutting specimen changed linearly with the external static load, and the fitting sensitivity is 0.0286 με/N. Combined with ANSYS finite element analysis, it is verified that the simulation analysis results are close to the measured data, which verifies the effectiveness and monitoring accuracy of the sensor. The dynamic load test results reflect the internal strain change trend of asphalt mixture under external rutting load, confirming that the encapsulated FBG sensor is suitable for the long-term monitoring of asphalt pavement strain.

## 1. Introduction

Asphalt pavement is the main structure type of domestic expressways. Because of its unique engineering advantages, it is very important in expressway construction [1]. Compared with other forms of roads, asphalt pavement has the advantages of convenient maintenance, shock absorption and noise reduction, rapid construction, and recycling. However, due to the influence of various unfavorable factors in the use of asphalt pavement, rutting, subsidence, cracks, and other diseases often occur [2]. Traditional pavement monitoring methods have problems such as low accuracy, low frequency, and strong destructiveness. It is difficult to achieve long-term and continuous monitoring [3], which makes it difficult to detect and deal with pavement diseases in time. Fiber grating sensors show great potential in the monitoring of asphalt pavement due to their advantages of anti-electromagnetic interference, corrosion resistance, and high sensitivity [4], which can provide accurate data support for pavement health assessment. However, in the actual use process, fiber Bragg grating (FBG) sensors have problems such as large differences in modulus from the pavement structure and weak shear resistance, which can easily cause measurement errors or sensor damage. Therefore, it is of great significance to use appropriate packaging materials to protect FBG sensors [5].

FBG sensors have shown superior performance in the field of structural monitoring, so they have been widely used in the field of civil engineering. Meng S et al. [6] proposed an implantable cantilever sensing beam based on FBG to monitor the permanent deformation of subgrade in seasonally frozen areas under vehicle load and freeze–thaw cycles, and verified its feasibility through three years of field tests. Liu et al. [7] tested the strain response of four kinds of base asphalt pavement under a 100 kN dynamic load based on FBG sensors and revealed the compression–tension–compression cycle in the surface layer and the tensile strain characteristics of the base layer. They found that reducing the stiffness of the cement stabilized the base layer and maintaining modulus continuity can optimize the strain distribution and improve the bearing capacity of pavement. Liao et al. [8,9] established a moving load identification model of asphalt pavement by combining the measured data of FBG sensors and a BP neural network, and verified the accuracy of this method for vehicle load identification by finite element simulation. Li Jianxin et al. [10] established a monitoring method for concrete crack propagation through FBG sensors and a plastic damage model, verified the quantitative relationship between damage factor and crack width, and provided an effective means for structural damage assessment. In summary, FBG sensors have been successfully applied to measure and monitor many factors, such as pavement strain, temperature, deformation, and damage, achieving significant results. However, there are still challenges in the embedding process, survival rate, accuracy, data demodulation technology, and other aspects of FBG sensors.

In order to ensure that the sensor is not damaged during the normal use of asphalt pavement, there are higher requirements for the strength, corrosion resistance, high temperature resistance, and wear resistance of the packaging material. Shi Q.Q. et al. [11,12,13] prepared highly oriented CNT/EP composites by a continuous impregnation stretching and step pressing process. The tensile strength and toughness of the CNT/EP composites were 3.5 GPa and 80.2 J cm^−3^, respectively, which are better than those of traditional CNT and carbon fiber-reinforced composites. Song X. et al. [14] modified carbon nanotubes (CNTs) on electrospun PCL nanofibers by ultrasonic assistance to construct a flexible interwoven structure of CF/EP laminates. After curing, PCL phase separation caused the CNTs to disperse uniformly while maintaining the glass transition temperature of the epoxy resin. Chen et al. [15] used amino-modified multi-walled carbon nanotubes and a low concentration of polyetherimide to synergistically toughen epoxy resin. After testing, the fracture toughness, impact strength, and bending strength of epoxy resin were significantly improved under the synergistic effect. Xin et al. [16,17,18] prepared epoxy resin composites using aligned multi-walled carbon nanotubes. The effects of different carbon nanotube contents on the mechanical, electrical, morphological, and fatigue properties of CNT/EP composites were studied, and the excellent mechanical durability of this composite was verified. Epoxy resin (EP) has excellent properties, such as high adhesion, temperature resistance, corrosion resistance, and processability after curing [19], but there are high brittleness defects. Carbon nanotubes (CNTs) are ideal reinforcement materials due to their unique hollow structure and high aspect ratio, high strength and high modulus [20]. In this paper, CNT-reinforced EP composites were selected as the packaging substrate for fiber Bragg grating (FBG) sensors. Zhong et al. [21] used three kinds of pressure-sensitive packaging materials, including polycarbonate, polyethylene, and epoxy resin. The pressure sensitivity is about 200 times higher than that of bare fiber grating, and the mechanical strength is high. Huang et al. [22] manufactured all-silicon sensors, using silicon glass for the substrate and packaging materials. The sensing principle and performance test of the tilt sensor are described in detail. The test results show that the packaged FBG has good packaging quality and sensing performance. Luo et al. [23] used epoxy resin as the adhesive material for sensor packaging and found that the smaller the thermal expansion coefficient of the packaging adhesive material, the smaller the influence on the output performance of the sensor in high-temperature environments.

In summary, FBG sensors provide a new technical means for asphalt pavement health monitoring by virtue of their anti-electromagnetic interference, corrosion resistance, high sensitivity, and other characteristics. However, they still face problems, such as mismatch with structural modulus and weak shear resistance in pavement applications, which can easily lead to measurement errors or sensor damage. EP has become a potential choice for FBG packaging materials due to its excellent adhesion, high temperature resistance, and chemical corrosion resistance, but its inherent brittleness limits its application in complex environments. CNTs can effectively enhance the mechanical properties of EP due to their high stiffness, high strength, and high aspect ratio. Therefore, the use of CNT/EP composites to encapsulate FBG sensors can not only effectively alleviate the stress concentration problem caused by the difference in modulus between the sensor and the pavement but can also enhance its shear resistance, fatigue resistance, and environmental adaptability, thereby improving the survival rate and monitoring reliability of the sensor in the high-temperature, high-load, and complex mechanical environments of asphalt pavement.

## 2. Results and Discussion

### 2.1. Analysis of Mechanical Properties

#### 2.1.1. Tensile Properties Analysis

**Figure 1:**
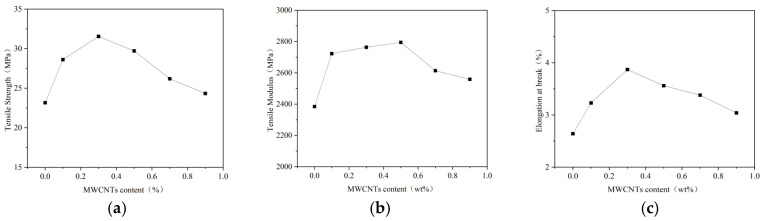
Tensile properties test results of composite materials. (**a**) Tensile strength of composite materials. (**b**) Tensile modulus of composites. (**c**) Fracture elongation of composite materials.

Figure 1a–c present the results of the tensile property tests of the composites.

It can be seen in the figure that under the condition of the same amount of epoxy resin, curing agent, and accelerator, the tensile strength, tensile modulus, and elongation at break of carboxylated carbon nanotubes/epoxy resin composites were improved by adding different amounts of carboxylated carbon nanotubes to the epoxy resin matrix. When the content of carboxylated carbon nanotubes was 0.3 wt%, the tensile strength and elongation at break of the composites reached 31.54 MPa and 3.87%, which were 36.2% and 47% higher than those of the pure epoxy resin, respectively. The tensile modulus of carboxylated carbon nanotubes/epoxy resin composites reached a maximum of 2794.5 MPa when the content of carbon nanotubes was 0.5 wt%, which was 17.2% higher than that without carbon nanotubes. It can be seen that with the change in the amount of carbon nanotubes added, the tensile strength, tensile modulus, and elongation at break of the composites show a trend of increasing first and then decreasing.

This phenomenon shows that when an appropriate amount of carbon nanotubes is added to epoxy resin, they can be uniformly dispersed throughout the material system. However, as the carbon nanotube content further increases beyond the critical value, the carbon nanotubes cannot continue to maintain a good dispersion state, resulting in uneven internal structure of the composite material, making it more prone to damage.

#### 2.1.2. Bending Performance Analysis

**Figure 2:**
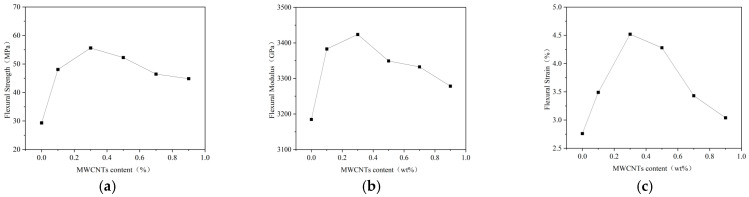
Bending test results of composite materials. (**a**) Bending strength of composite materials. (**b**) Bending modulus of composite materials. (**c**) Bending strain of composite materials.

Figure 2a–c show the results of composite materials in bending tests.

It can be seen that the addition of carbon nanotubes (CNTs) to epoxy resin matrix can significantly improve the flexural properties of composites. When the CNT content was 0.3 wt%, the flexural strength (55.61 MPa), flexural modulus (3423.6 MPa), and flexural strain (4.52%) of the carboxylated CNT/epoxy resin composites reached the maximum values, which were 89.7%, 7.5%, and 63.8% higher than those of pure epoxy resin, respectively. The improvement in the flexural strength and flexural strain was particularly significant. When the CNT content exceeded 0.3 wt%, the flexural properties of the composites began to decrease, indicating that the CNTs agglomerated, affecting the properties.

Tensile and flexural properties tests showed that the addition of 0.3 wt% CNT optimized the mechanical properties of epoxy resin composites. Further increases in CNT content will lead to a decrease in performance. It is confirmed that 0.3 wt% is the best addition ratio, which can not only give full play to the enhancement effect of CNTs but can also avoid the problem of agglomeration and uneven dispersion caused by excessive addition.

### 2.2. Infrared Spectrum Analysis of Carboxylated Carbon Nanotubes

**Figure 3:**
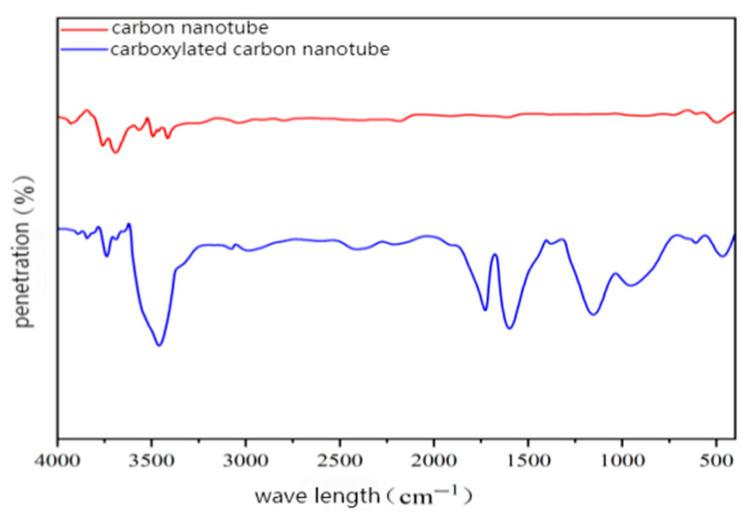
Infrared spectra of carbon nanotubes before and after modification.

The multi-walled carbon nanotubes and carboxylated multi-walled carbon nanotubes were studied by a wavenumber–transmittance test. The data obtained from the test were summarized into the infrared spectrum, and then the characteristic peaks were compared and analyzed. The test results are shown in Figure 3.

It can be seen in Figure 3 that the infrared spectrum of pure carbon nanotubes shows a stretching vibration peak of the O-H bond in the range of 3500 cm^−1^ to 3700 cm^−1^. This may be due to the fact that the surface of untreated carbon nanotubes contains trace impurities containing O-H groups or has adsorbed water from the air. Comparing the infrared spectra of carbon nanotubes before and after carboxylation, new absorption peaks were observed near 1200 cm^−1^, 1600 cm^−1^, and 1700 cm^−1^. These characteristic peaks correspond to the stretching vibration of the C-O bond, the bending vibration of the O-H bond, and the stretching vibration of the C=O bond in the carboxyl group, respectively, which indicates that the carboxyl (COOH) group is formed on the surface of the carbon nanotubes after carboxylation treatment. In addition, an obvious absorption peak was observed at about 3450 cm^−1^, where the absorption peak is related to the stretching vibration of the O-H bond, indicating the presence of hydroxyl (OH) or water molecules on the surface. These new chemical groups change the surface properties of carbon nanotubes, enabling them to form stronger chemical bonding or physical adsorption with epoxy resin, thereby improving the interfacial bonding strength between carbon nanotubes and epoxy resin.

### 2.3. SEM Micro-Morphology Characterization Analysis

#### 2.3.1. Morphology Characterization Analysis of Carbon Nanotubes Before and After Modification

**Figure 4:**
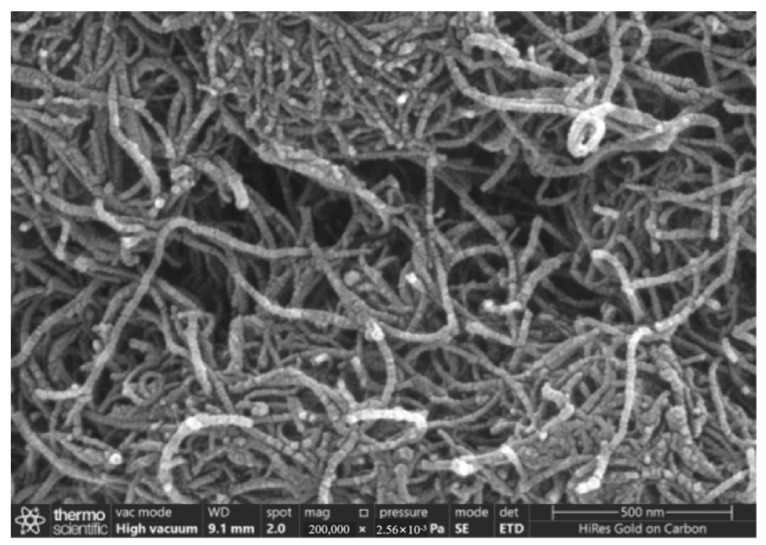
Unprocessed carbon nanotubes, magnified 200,000 times.

**Figure 5:**
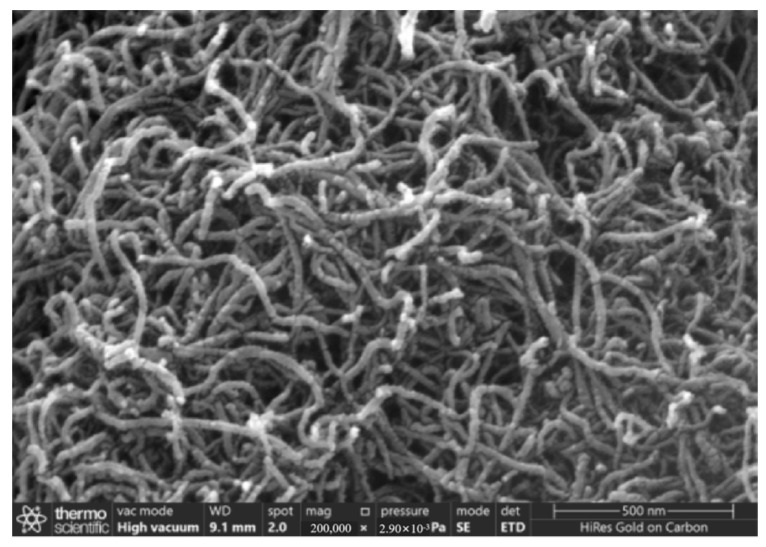
Carboxylated carbon nanotubes, magnified 200,000 times.

Figure 4 and Figure 5 present SEM photos of the untreated multi-walled carbon nanotubes and carboxylated multi-walled carbon nanotubes, magnified 200,000 times.

Scanning electron microscopy (SEM) observations showed that the surface of the unmodified carbon nanotubes was smooth and the tube wall structure was complete and tightly entangled into a network structure. The surface of the carboxylated carbon nanotubes was fuzzy and rough, the entanglement was reduced, and the arrangement was loose. This was due to the surface defects induced by mixed acid treatment, which provided grafting sites for -COOH/-OH groups. The introduction of surface groups enhances the polarity of carbon nanotubes and effectively weakens the interactions between the tubes through electrostatic repulsion and hydrogen bonding, thereby reducing entanglement. This indicates that carboxylation treatment can significantly reduce the agglomeration tendency of carbon nanotubes, improve their dispersion, and provide higher surface activity.

#### 2.3.2. Analysis of Tensile Fracture Surface Morphology of Composites

**Figure 6:**
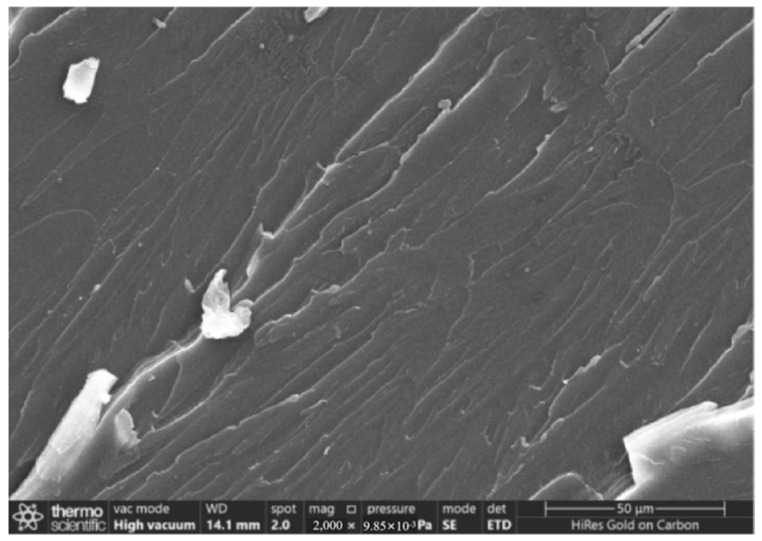
Tensile fracture surface of pure epoxy resin.

**Figure 7:**
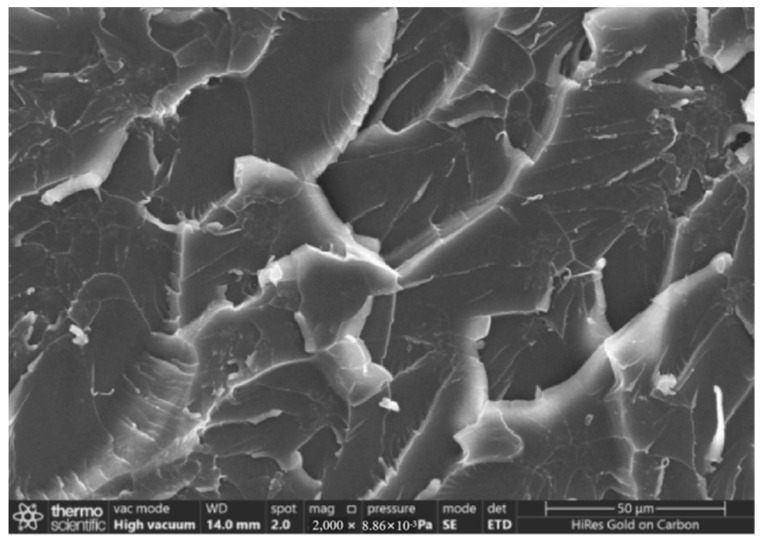
Tensile fracture surface of composite materials.

Figure 6 and Figure 7 present SEM diagrams of the tensile fracture surfaces of pure epoxy resin and carboxylated carbon nanotube/epoxy resin composites at the optimum content.

According to the observation results of scanning electron microscopy shown in Figure 6, at magnification of 2000 times, the tensile fracture surface of the pure epoxy resin shows a typical brittle fracture morphology. The fracture surface is relatively smooth and flat, the crack propagation path shows an obvious linear arrangement, and there are fewer crack branches. This morphological feature shows that under the tensile load, the microcracks inside the pure epoxy resin expand rapidly and connect with each other, and sudden fracture occurs almost without an obvious plastic deformation stage.

In contrast, as shown in Figure 7, at the same magnification, the tensile fracture surface of the composite material is rougher, and there are a large number of uneven tearing and plastic deformation traces. At the same time, the crack propagation path is tortuous and complex, and there are secondary cracks with radial distribution. These phenomena indicate that the composite material undergoes an obvious plastic deformation stage before fracture. This change is likely due to the introduction of carbon nanotubes, which leads to the effective transmission and dispersion of external stress. When the epoxy resin matrix initially cracks under the action of external force, CNTs, as a nano-scale reinforcing material, can hinder the direct propagation of cracks, forcing cracks to deflect or bifurcate, thereby extending the crack propagation path. This indicates that the addition of carbon nanotubes can not only effectively bear the external applied force but can also absorb the energy in the fracture process, thus significantly improving the tensile properties and toughness of the composites.

### 2.4. Calibration Test Analysis

#### 2.4.1. Analysis of Strain Calibration Test

**Figure 8:**
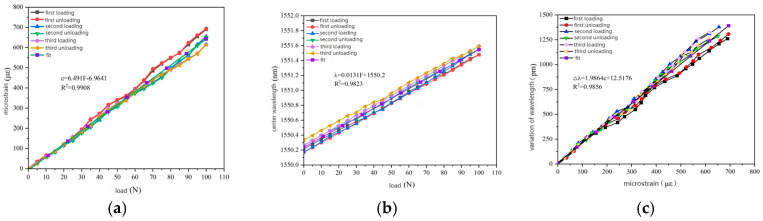
Strain calibration test results. (**a**) The relationship between load and micro-strain. (**b**) The relationship between load and center wavelength. (**c**) The relationship between micro-strain and wavelength change.

Since the measured experimental data are discrete, the least squares fitting method was used to fit the data. The fitting curves are shown in Figure 8a–c, including the relationship between load and micro-strain, the relationship between load and central wavelength, and the relationship between micro-strain and wavelength change.

It can be seen in Figure 8 that the fitted relationships between load and micro-strain, load and center wavelength, micro-strain and wavelength change are 6.494 μm/N, 0.0131 nm/N, and 1.9864 pm/μm, respectively. The correlation coefficient exceeds 0.98. This indicates that the packaging material exhibits good elastic behavior, good stability, and repeatability during loading and unloading. By analyzing the three loading and unloading processes, it can be concluded that during the initial loading and unloading process, the change in the micro-strain of the packaging material is not completely linear, and there may be some residual strains. However, as the number of loading and unloading steps increases, the behavior of the packaging material becomes gradually stable.

As presented in Section 2.3, the theoretical value of the axial strain sensitivity coefficient of the bare fiber is 1.22 pm/μm. The strain sensitivity coefficient of the packaged FBG sensor reaches 1.9864 pm/μm. This means that the strain sensitivity of the encapsulated sensor is about 1.63 times higher than that of the bare fiber, significantly enhancing the sensitivity of strain monitoring.

#### 2.4.2. Temperature Calibration Test Analysis

**Figure 9:**
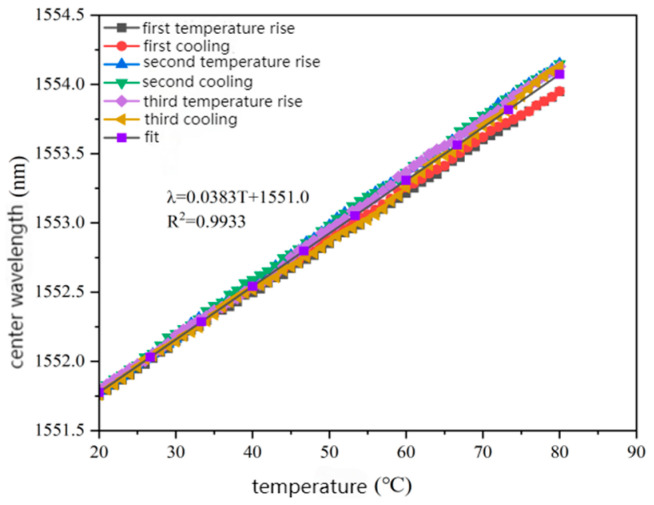
The relationship between temperature and central wavelength.

The least square method was used to fit the calibration test data of three heating and cooling cycles, and the fitting curve of the center wavelength of the sensor with the change of temperature was obtained, as shown in Figure 9.

It can be seen in Figure 9 that the encapsulation of fiber grating by carboxylated carbon nanotube/epoxy resin composite material will not destroy the temperature sensing characteristics of the sensor. The encapsulated sensor shows good thermal stability and repeatability after multiple heating and cooling cycles. This is suitable for the application scenario of asphalt pavement, which requires long-term temperature monitoring. After multiple temperature cycles, the fitted correlation coefficient exceeded 0.99, indicating that the sensor has good linearity. The temperature sensitivity of the sensor was 0.0383 nm/°C, which is 3.61 times higher than the theoretical temperature sensitivity coefficient of bare fiber of 10.6 pm/°C, calculated in the second chapter. In the temperature cycle test of 20 °C → 80 °C → 20 °C, the change in the center wavelength of the sensor was strictly linear with the temperature (R2 > 0.99). The temperature sensitivity was 0.0383 nm/°C, and the three cycle data overlapped, with no thermal hysteresis phenomenon and stable performance in a high-temperature environment.

### 2.5. Applicability Analysis of the Sensor

#### 2.5.1. Static Load Test Analysis

**Figure 10:**
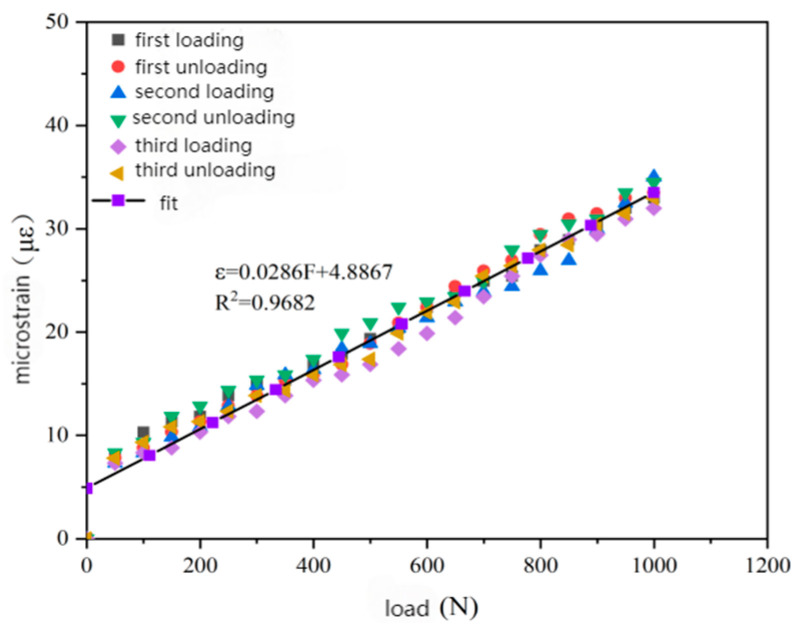
Relationship between load and strain in static load test.

In the static load test of the rutting specimen, according to the change in the central wavelength, the strain change in the corresponding load at the sensor embedding position was calculated by the fitting curve formula shown in Figure 10. The relationship curve between the external load and the calculated strain was obtained by the least square fitting of the three loading and unloading cycle data, as shown in Figure 10.

According to the fitting results in the Figure 10, it can be seen that there is a linear relationship between the strain change in the internal structure of the asphalt concrete rut specimen detected by the FBG sensor and the change in the external static load. With the adjustment of the applied static load, the internal strain monitored by the sensor shows a linear change trend accordingly. The load/shear force direction indirectly changes the sensitivity of the sensor by affecting the stress distribution of the packaging material, the interface bonding state, and the enhancement efficiency of CNTs. The static load test verified that the strain transfer efficiency was high under vertical load. The horizontal shear load may lead to a decrease in sensitivity due to interface defects.

Based on the results of the static load test, it can be seen that the linear fitting sensitivity of the applied external static load and the internal structural strain of the rutting specimen monitored by the fiber Bragg grating demodulator was 0.0286 με/N, and the correlation coefficient was R2 = 0.9682, indicating that the linear relationship is good. With the increase in external load, the strain of the sensor buried position also increased, which can well reflect the strain change inside the asphalt mixture rutting specimen.

#### 2.5.2. ANSYS Finite Element Analysis

(1) Build models and set attributes

A model was established according to the actual size of the specimen. The size of the rutting specimen model was 300 mm × 300 mm × 50 mm, and the size of the sensor model was 10 mm × 10 mm × 100 mm. Because only the analysis and calculation of strain were carried out, only the elastic modulus and Poisson’s ratio of the material were defined. According to the specification requirements, the elastic modulus of AC-13 asphalt mixture material is 1400 MPa, and the Poisson’s ratio is 0.25; the elastic modulus of carbon nanotube/epoxy resin composites is 2800 MPa, and the Poisson’s ratio is 0.35.

(2) Meshing and adding load

**Figure 11:**
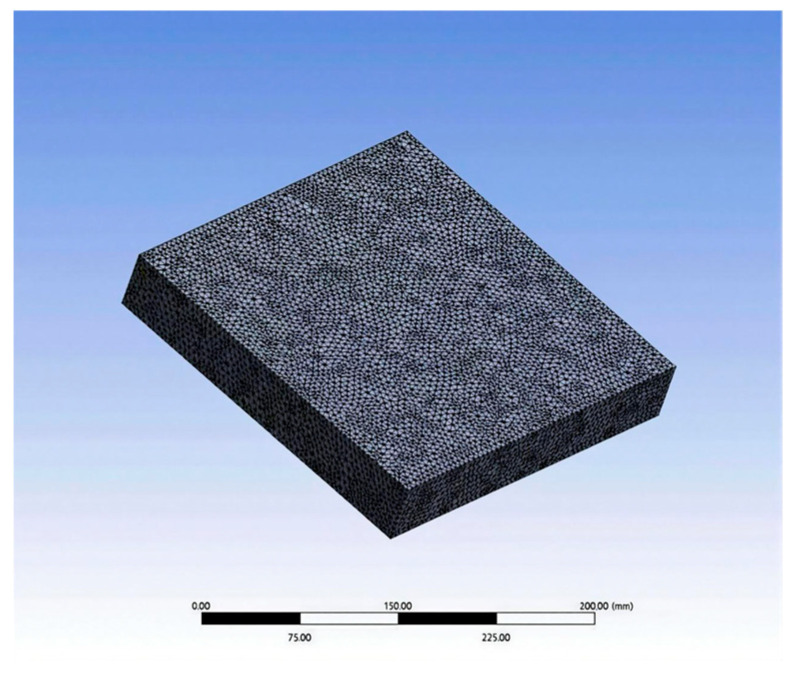
Rutting specimen model.

**Figure 12:**
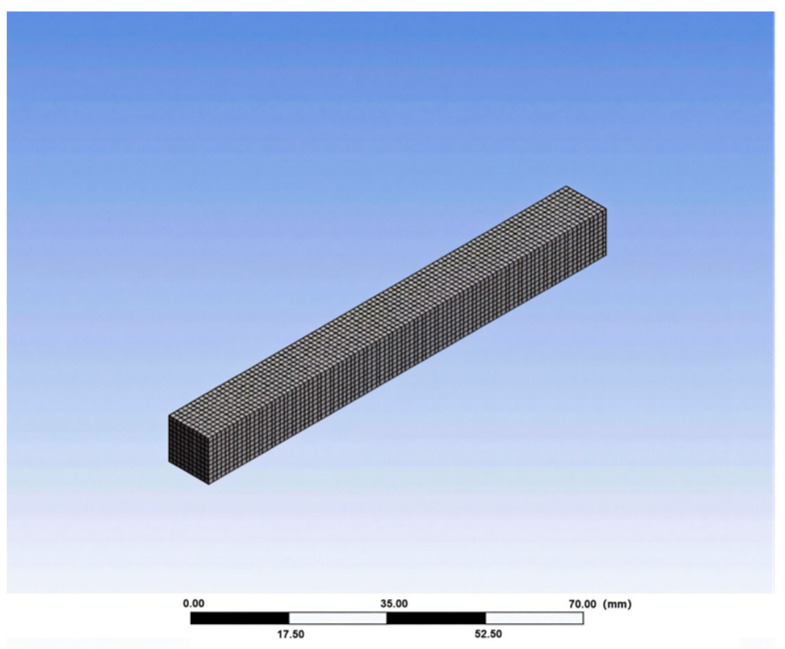
Sensor model.

The rut specimen model was meshed by SOLID186 ten-node tetrahedron, and the unit size was set to 5 mm. The sensor model was meshed by SOLID186 twenty-node hexahedron. In order to make the calculation result more accurate, the sensor model mesh was encrypted, and the unit size was 1 mm. The finite element model is shown in Figure 11 and Figure 12. Fixed loads were added to the four sides of the model and the bottom surface. The static load was arranged at the center of the upper surface of the model as a circular uniform load with a diameter of 150 mm.

(3) Solution analysis

**Figure 13:**
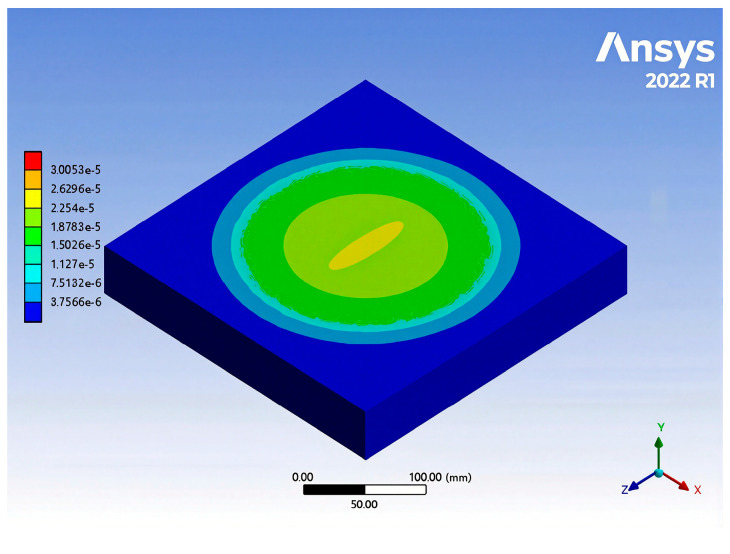
Strain distribution cloud map.

The circular uniform load was added to the upper surface of the asphalt mixture rutting test piece, the load of each stage was set to 49.98 N, and the load was applied cumulatively over 20 cycles. A strain cloud diagram was obtained after the finite element calculation, as shown in Figure 13.

**Figure 14:**
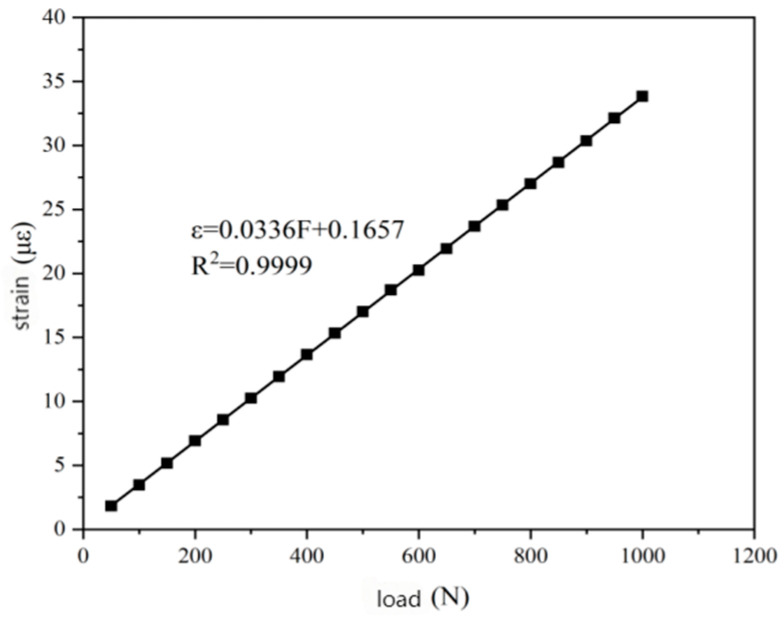
The relationship between load and strain.

Origin 2021 software was used to draw a scatter plot of the obtained data, and the data were fitted by the least squares method to obtain the fitting curve of the load and strain in ANSYS finite element simulation analysis, as shown in Figure 14.

It can be seen in Figure 14 that the slope between the strain and load of the asphalt mixture rutting specimen simulated by finite element analysis was 0.0336 με/N under static load, and the correlation coefficient exceeded 0.99. However, compared with the slope of the fitting curve of 0.0286 με/N in the static load test, there is a certain deviation. Therefore, the strain sensing coefficient θ was introduced, and θ = 0.85 was obtained by comparing the measured value with the theoretical value. This deviation may be caused by many factors. Firstly, during the fabrication of the sensor, the surface of the fiber grating may not have been completely clean, resulting in insufficient curing and bonding with the epoxy resin matrix, leading to slight slip between the two. Secondly, the epoxy resin and curing agent inevitably produced bubbles during the heating and curing process. Although the defoamer can inhibit the formation of bubbles, there is still a small amount of residue. In addition, in the ANSYS finite element simulation, it was assumed that the sensor and the asphalt mixture were completely bonded, and there may have been a small gap between the two in the actual situation. These factors may have led to errors in the monitoring process of the sensor, which in turn affected the sensitivity of the sensor. Under the action of external load, the stress distribution of the high-aspect-ratio device is more uniform along the length direction, which can reduce the end effect and improve the strain transfer efficiency. The low-aspect-ratio device has relatively stronger compressive and shear resistance, and is more suitable for high-stress areas.

Although there was a certain error between the finite element simulation and the actual test, the response trends of the two were basically the same, which verifies the effectiveness of the sensor and the accuracy of the monitoring process. In general, the actual test results can better reflect the strain response law of asphalt mixture under static load, and the finite element simulation further verifies the reliability of sensor detection. Although there were problems such as fiber grating surface cleanliness, bubble residue during epoxy resin curing, and imperfect interface bonding between the sensor and the asphalt mixture, these factors did not significantly affect the overall performance of the sensor.

#### 2.5.3. Dynamic Load Test Analysis

**Figure 15:**
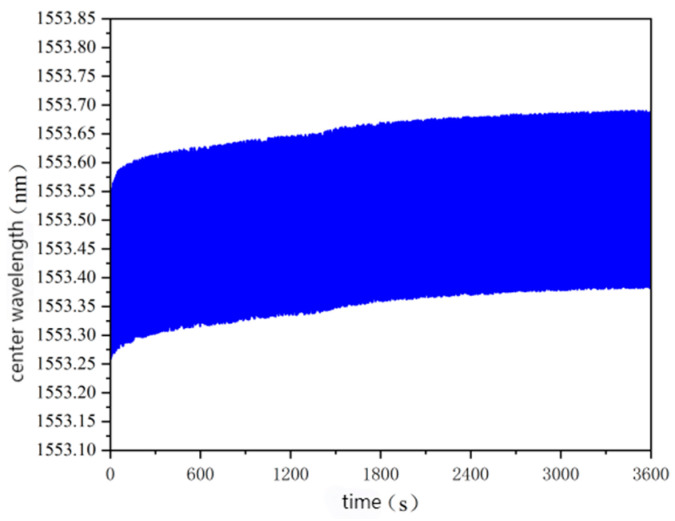
Diagram of the sensor wavelength changes in the rutting test.

A dynamic load rutting test was used to simulate the dynamic load of asphalt pavement with driving vehicles, and the performance of the sensor under dynamic load was evaluated. The rutting test was carried out for 1 h, and the internal strain of the specimen under the action of dynamic load changed. According to the collected data, the waveform is shown in Figure 15.

In the image, it can be seen that after the 1h rutting dynamic load test, the central wavelength change range of the sensor was about 430 pm. The internal strain of the corresponding asphalt concrete structure was calculated by the fitting curve formula in Figure 14. It was about 210 με.

Because of the large amount of data, it is difficult to see the change trend, so the difference between the peak value and the initial wavelength was used for plotting, and the relationship curve between the difference and the time change is shown in Figure 11.

**Figure 16:**
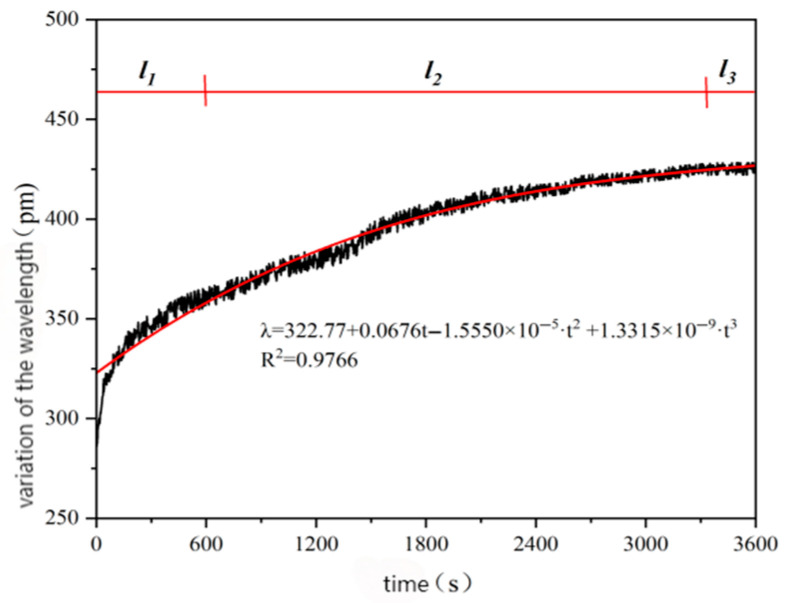
The relationship between wavelength change and time.

It can be seen in Figure 16 that the relationship between the change in the center wavelength of the sensor and the time in the rutting test follows a cubic polynomial change rule. It can be observed that the coefficient of the monomial is positive, which indicates that the wavelength variation λ shows an increasing trend with the increase in the test time t in the initial stage, and there is an obvious growth period. However, due to the negative coefficient of the quadratic term, the growth rate of the wavelength variation gradually slows down over time, eventually leveling off. The coefficient of the cubic term is very small, and the impact on the overall trend can be ignored.

Therefore, Figure 16 is roughly divided into three stages. In the l_1_ (0–600 s) stage, the central wavelength changes significantly and the increase is large. Subsequently, with the progress of the rutting test, it can be seen that the change in the center wavelength of the sensor in the l_2_ (600–3300 s) stage shows a steady upward trend, and the wavelength change in the l_3_ (3300–3600 s) stage gradually levels off, with a small increase, almost reaching a stable state, indicating that the wavelength change has basically stagnated and there is no obvious fluctuation. The sensor can still work stably under multi-directional complex load environment and meet the long-term monitoring requirements of asphalt pavement, but the packaging process needs to be optimized to reduce the direction dependence.

The change trend of the central wavelength of these three stages reflects the internal strain change trend of asphalt concrete under the action of external rutting load, which is of great significance for studying the internal structure response law of asphalt concrete. After an hour-long rutting test, the sensor showed good working performance and no damage, meeting the requirements of long-term monitoring of asphalt pavement strain. The results show that the strain monitored by the sensor was highly linear with the load, and the fitting sensitivity was 0.0286 με/N (R2 = 0.9682). In the dynamic load test of simulated vehicle wheel load (70 MPa of tire pressure, 42 times/min rolling) for 1 h, the center wavelength variation range was only 430 pm (corresponding to strain 210 με), and there was no abnormal fluctuation or signal interruption, indicating that the stability under load was good.

## 3. Conclusions

(1) When the content of carboxylated carbon nanotubes (CNTs) in epoxy resin (EP) was 0.3 wt%, the tensile and flexural properties of CNT/EP composites were optimal. When the content exceeded this amount, the performance showed a downward trend, indicating that 0.3 wt% is the best content. Infrared spectroscopy confirmed that carboxyl groups were successfully grafted on the surface of CNTs by the mixed acid reflux method, and the characteristic absorption peaks were located at 1200 cm^−1^, 1600 cm^−1^, 1700 cm^−1^, and 3450 cm^−1^, respectively. This modification significantly improved the dispersion and interfacial bonding strength of CNTs in EP.
(2) According to scanning electron microscopy analysis, after carboxylation treatment, the agglomeration of carbon nanotubes was significantly reduced, the size of the aggregates was significantly reduced, the degree of entanglement was reduced, the arrangement between carbon nanotubes was relatively loose, and the dispersion was improved. Comparative analysis of the microstructure of the tensile fracture surface of the material was carried out. The tensile fracture surface of the composite material was rougher, and there was a large number of uneven tears and plastic deformation traces. The crack propagation path was tortuous and complex, and radial secondary cracks appeared. This indicates that the addition of carbon nanotubes can effectively bear the external applied force, absorb the energy in the fracture process, and enhance the toughness of the material.
(3) Through the calibration test of the sensor, it can be concluded that the packaging material has good elastic behavior, good stability, and repeatability when subjected to external load and temperature changes. The strain sensitivity coefficient of the packaged FBG sensor reached 1.9864 pm/μm, which is about 1.63 times higher than that of the bare fiber. The temperature sensitivity of the sensor was 0.0383 nm/°C, which is an increase of 3.61-fold.
(4) According to the analysis of the applicability of the sensor, under a certain amount of static load, the internal strain of the asphalt concrete specimen monitored by the sensor showed a corresponding linear change trend. The response trends of finite element simulation and the static load test were basically the same, verifying the effectiveness of the sensor and the accuracy of the monitoring process. Under the action of simulated wheel load, the relationship between the change in the center wavelength of the sensor and the time shows a cubic polynomial change rule, reflecting the internal strain change trend of asphalt concrete under the conditions of external wheel load.

## 4. Materials and Methods

### 4.1. Carbon Nanotubes

In this paper, carbon nanotubes were selected as multi-walled carbon nanotubes (MWCNTs) produced by Suzhou Carbonfeng Graphene Technology Co., Ltd. (Suzhou, China). The carbon nanotubes have high purity, a large aspect ratio, and a high specific surface area, and the comprehensive advantages are obvious. Multi-walled carbon nanotubes with a purity of >95 wt%, an inner diameter of 3–5 nm, an outer diameter of 8–15 nm, and a length of 3–12 μm were used.

### 4.2. Epoxy Resin

The gel material used in this paper was bisphenol A epoxy resin, which is a widely used high-performance thermosetting gel material. It is widely used because of its excellent mechanical properties, good adhesion, and chemical resistance. In order to achieve effective curing of epoxy resin, methyl tetrahydrophthalic anhydride (MeTHPA) was selected as the curing agent. MeTHPA is an efficient anhydride curing agent. Epoxy resin cured by anhydride curing agent usually shows high temperature resistance and mechanical properties, which is more suitable for the high-temperature environment of the asphalt pavement construction process. However, the reaction rate of MeTHPA at lower temperatures is slow, which prolongs the time required for curing and requires a higher curing temperature to complete the curing reaction. In order to solve this problem, 2,4,6-tris (dimethylaminomethyl) phenol (DMP-30) was added as an accelerator to accelerate the curing rate, allowing the curing process to occur at a relatively low temperature. In addition, defoamer, release agent, and acetone solution were also used in the experiment.

### 4.3. Fiber Grating

The bare fiber grating selected in this paper was produced by Jinan Dahui Photoelectric Technology Co., Ltd. (Jinan, China). It has a bare fiber grating center wavelength of 1550 ± 0.5 nm, reflectivity ≥90%, bandwidth ≤0.3 nm, and a grating area length of 10 mm.

Combined with the photosensitive characteristics of the fiber material and the sensing principle of the fiber grating, the theoretical value of the axial strain sensitivity coefficient of the quartz fiber was roughly calculated to be 1.22 pm/με, and the theoretical value of the temperature sensitivity coefficient was 10.6 pm/°C.

### 4.4. Preparation of Carboxylated Carbon Nanotubes

**Figure 17:**
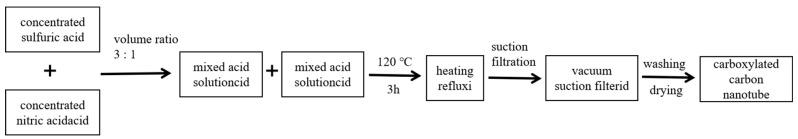
Flowchart of carboxylated carbon nanotubes by acid reflux.

Due to the strong van der Waals force between carbon nanotubes, they tend to attract each other and gather together to form clusters or bundle structures, which are difficult to uniformly disperse in a polymer matrix. This agglomeration phenomenon causes the actual performance of the prepared carbon nanotube-modified gel material to fail to meet the theoretical expectations, and may even cause a small increase in strength or a decline in performance. Therefore, in this paper, the carboxyl functionalization of carbon nanotubes was carried out by the mixed acid reflux method. The modification process is shown in Figure 17. The specific steps are as follows:

(1) The mixed acid solution of concentrated sulfuric acid (H_2_SO_4_) and concentrated nitric acid (HNO_3_) was prepared according to a volume ratio of 3:1.
(2) A certain amount of MWCNTs was placed in a clean beaker, and enough mixed acid solution was slowly added to ensure that all carbon nanotubes were completely immersed. A glass rod was used to gently stir the carbon nanotubes in the mixed acid along the wall of the beaker to initially break up the possible aggregates and ensure that the acid solution fully contacted the surface of the carbon nanotubes.
(3) The whole mixture was carefully transferred into a round-bottomed flask, and the temperature was controlled by a constant-temperature heating sleeve. The mixture was continuously heated and refluxed at 120 °C for 3 h until the carbon nanotubes were fully oxidized.
(4) After the reaction system was naturally cooled to room temperature, the reaction solution was diluted with deionized water to reduce the concentration of strong acid. The diluted solution was subjected to multiple suction filtration using a vacuum suction filtration device until a pure carboxylated carbon nanotube was obtained.
(5) In order to ensure that the residual acid was completely removed, the modified carbon nanotubes needed to be washed repeatedly with deionized water again until the carbon nanotubes were neutral. Then, the cleaned carboxylated carbon nanotubes were transferred to the preheated oven for drying treatment.

### 4.5. Preparation of CNT-Modified Gel Materials

**Figure 18:**
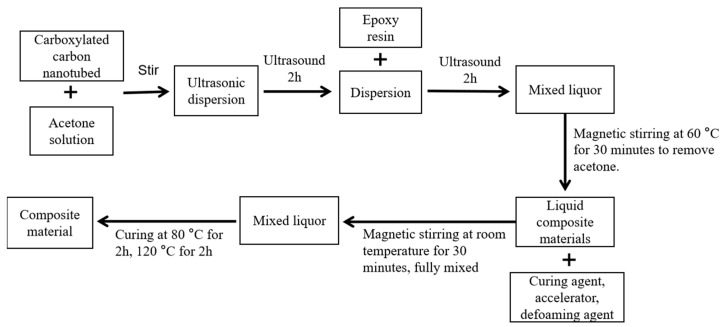
Preparation process of carboxylated carbon nanotubes/epoxy resin composites.

In order to achieve uniform dispersion of carbon nanotubes, the modified gel material was prepared by ultrasonic dispersion. The preparation process is shown in Figure 18.

The specific steps were as follows:

(1) Carbon nanotubes with different mass percentages were added to acetone solution and treated with an ultrasonic disperser for 2 h.
(2) Epoxy resin was added to the dispersed solution according to the predetermined ratio, and ultrasonic dispersion was performed again for 2 h.
(3) The acetone solvent in the system was removed by magnetic stirring for 30 min at 60 °C. After the mixture cooled to room temperature, the anhydride curing agent was added at a mass ratio of 100:80 (epoxy resin/curing agent), and 0.5% defoaming agent and DMP-30 accelerator were added at the same time.
(4) Continuing magnetic stirring was performed for 30 min to ensure that all components were fully and evenly mixed. Then, the prepared mixed liquid was poured into the mold. According to the specific curing process, it was cured at 80 °C for 2 h, and then raised to 120 °C for 2 h to finally obtain the carboxylated carbon nanotube-reinforced epoxy resin composite sample.

### 4.6. Tensile and Bending Tests

**Figure 19:**
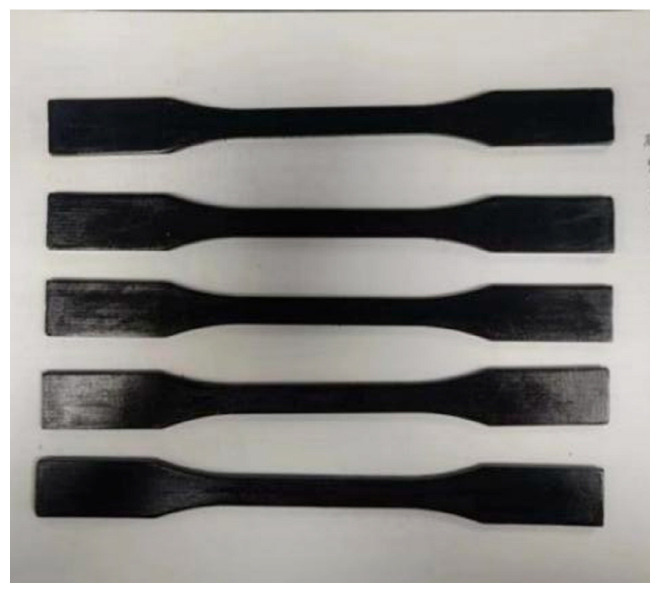
Carbon nanotubes/epoxy resin tensile specimens.

**Figure 20:**
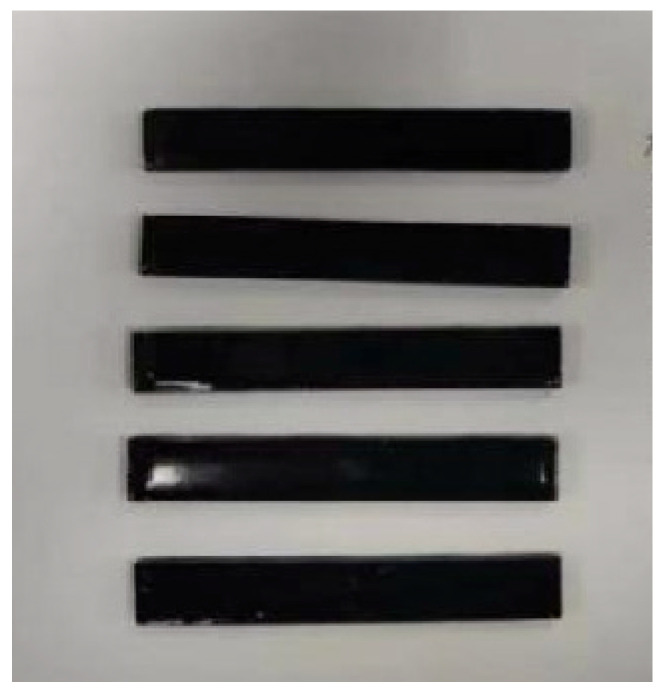
Carbon nanotube/epoxy resin bending specimens.

In order to explore the optimal content of carboxylated multi-walled carbon nanotubes in epoxy resin, six groups of proportions were designed, and the contents of each group were 0.0%, 0.1%, 0.3%, 0.5%, 0.7%, and 0.9%, respectively. Tensile specimens and bending specimens of composite materials were prepared according to GB/T 1040.1-2018 [24] and GB/T 9341-2008 [25], respectively. The specimen is shown in Figure 19 and Figure 20.

The tensile and bending properties of the prepared carboxylated carbon nanotube/epoxy resin composite specimens were tested by a microcomputer-controlled electronic universal testing machine (WDW-50C). When measuring the tensile strength, the loading rate was set to 10 mm/min, and then to 2 mm/min when measuring the elongation at break, tensile modulus, bending strength, bending modulus, and bending strain.

### 4.7. Fourier Transform Infrared Spectroscopy Test

In the experiment, FTIR was used to analyze the functional groups on the surface and edge of the prepared carboxylated carbon nanotubes, and the changes in the functional groups of the carbon nanotubes before and after modification were compared and observed. The instrument used a Thermo Nicolet IS5 infrared spectrometer from the Thermo Nicolet Technology Company (Waltham, MA, USA). The test range was 400 cm^−1^ to 4000 cm^−1^, the resolution was 4 cm^−1^, and the number of scans was 32.

### 4.8. Scanning Electron Microscope Test

The effect of the carboxylation process on the dispersion of carbon nanotubes was observed by comparing the original multi-walled carbon nanotubes without mixed acid treatment with the carboxylated multi-walled carbon nanotubes using the Zeiss 300 scanning electron microscope produced by German Carl’s company (Wuppertal, Germany). A comparative SEM experiment was conducted on the tensile section of the tensile specimen of pure epoxy resin and the tensile specimen of the composite material with the best content of carboxylated carbon nanotubes to observe the microstructure of the fracture surface. In order to obtain a clearer image and reduce the charging effect, all samples were sprayed with gold.

### 4.9. Packaging of Fiber Grating Sensor

In order to improve the durability and protection of bare fiber in asphalt pavement construction, multi-level protection measures were taken for bare fiber gratings. The steps were as follows:

(1) Hytrel is a thermoplastic polyester elastomer with excellent wear resistance, chemical corrosion resistance and fatigue resistance. It can effectively protect optical fibers from the influence of the external environment and ensure that they are tightly and safely wrapped.
(2) The Hytrel loose sleeves with optical fibers are further encapsulated into armored sleeves with an inner diameter of 1.1 mm. The armored sleeves are equipped with a stainless steel protective sleeve layer, which can effectively protect the optical fiber from damage in the asphalt pavement.
(3) The FBG sensor is protected without affecting the accuracy of the sensor by encapsulating the fiber grating area with CNT/EP composites.

**Figure 21:**
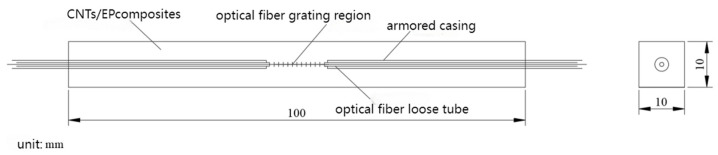
Package structure diagram of the FBG sensor.

The sensor package structure was designed as a cuboid shape. In order to avoid the adverse effect of excessive cross-section size on the stress and strain distribution of the structure, the cross-section size was determined to be 10 mm × 10 mm × 100 mm. The sensor package structure is shown in Figure 21.

**Figure 22:**
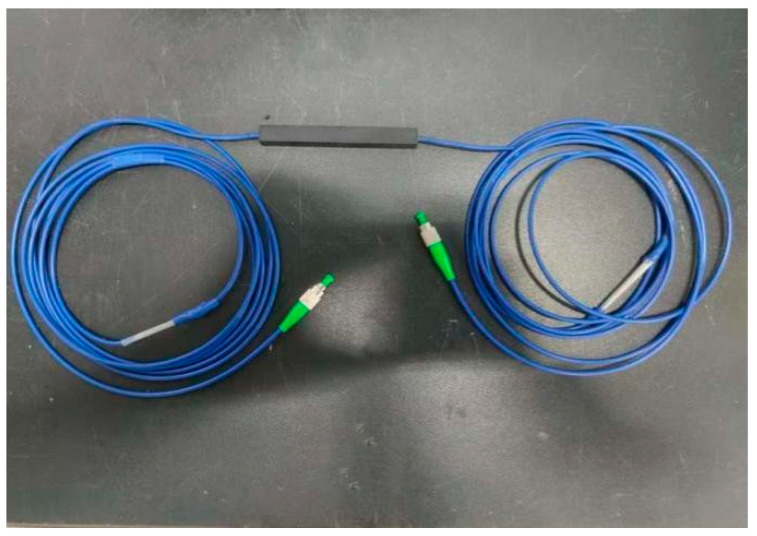
Physical diagram of the sensor.

Using a special steel mold, the fiber grating with armored casing protection was inserted into the mold, and the carbon nanotube/epoxy resin composite mixture was injected. The gradient curing process (80 °C and 120 °C for 2 h each) was used to complete the curing. After demolding, the fiber grating was cured in a standard environment for 24 h. Finally, the fiber grating was fused with the jumper by a fiber splicer to prepare a complete FBG sensor. The fabricated FBG sensor is shown in Figure 22.

### 4.10. Sensor Calibration Test

Although the developed package provides the necessary physical protection for the fiber grating, it changes the stress and temperature sensing characteristics of the grating, affecting its sensing performance. In order to ensure that the sensor can accurately measure strain in practical applications, it must be calibrated before use. Strain sensitivity calibration was performed at room temperature using a microcomputer-controlled electronic universal testing machine. Tensile force from 0 N to 100 N was applied to the sensor, and the center wavelength change was recorded every 5 N increase using the fiber grating demodulator. The resistance strain gauge was pasted on the surface of the sensor, and the strain change under the stress value was measured by the strain gauge. By comparing and analyzing the strain change and the center wavelength drift data of the fiber grating, the relationship curve between the center wavelength change and the strain was drawn, and the sensitivity of the sensor was finally determined to realize the strain calibration of the sensor. The temperature calibration test was carried out by the oven method. The initial temperature was 20 °C, and the final temperature was increased to 80 °C, and then the temperature was gradually decreased. During the whole process, data acquisition was carried out for every 1 °C change. In order to ensure the accuracy of the test, the strain and temperature calibration test was repeated for three cycles.

### 4.11. Sensor Adaptability Analysis Test

The prepared sensor was embedded in an AC-13 asphalt rutting specimen to simulate the actual embedding of the sensor in asphalt pavement. Through static load and dynamic load testing of the asphalt rutting specimen with the embedded sensor, the validity and accuracy of the sensor in the asphalt pavement were verified. The test used #70 matrix asphalt, and its performance meet the requirements of specification JTG E20-2011 [26]. The relevant performance indicators of each grade aggregate and mineral powder meet the requirements of JTG 3432-2024 [27].

The static load test adopted the method of step-by-step loading of heavy weights. In the experiment, the weight of the round cake was 5.1 kg, that is, each weight was 49.98 N. After connecting the optical fiber jumper and the optical fiber demodulator, the weights were placed in the center of the rut specimen in turn. A total of 20 weights were loaded, with a total of 999.6 N. After the loading was completed, they were unloaded one by one, and the loading and unloading cycles were performed three times.

In order to verify the accuracy of the sensor detection, ANSYS Workbench 2022 finite element software was used to simulate and analyze the static load test process, and the strain of the rut specimen under local static load was calculated. The whole process was roughly divided into the following three steps: (1) establishing the model and setting the attributes; (2) meshing and adding load; (3) performing solution analysis.

The dynamic load rutting test was carried out with reference to JTG E20-201126, and the change in the internal strain of asphalt mixture under a dynamic automobile wheel load was simulated. The test wheel was a rubber with a width of 50 mm and a tire pressure of 70 MPa ± 0.05 MPa. After 5 h of heat preservation in a 60 °C constant-temperature room, the specimens were rolled back and forth at a rolling speed of 42 times/min ± 1 times/min, and the central wavelength change during the rolling process was recorded by a fiber grating demodulator.

## Data Availability

Data are contained within the article. Some or all of the data, models, or code that support the findings of this study are available from the corresponding author upon reasonable request.
